# Hydrophobic Cellulose-Based Sorbents for Oil/Water Separation †

**DOI:** 10.3390/molecules29194661

**Published:** 2024-09-30

**Authors:** Karolina Tomkowiak, Bartłomiej Mazela, Zuzanna Szubert, Waldemar Perdoch

**Affiliations:** Faculty of Forestry and Wood Technology, Poznań University of Life Sciences, Wojska Polskiego 28, 60-637 Poznań, Poland; bartlomiej.mazela@up.poznan.pl (B.M.); zuzanna.szubert@up.poznan.pl (Z.S.); waldemar.perdoch@up.poznan.pl (W.P.)

**Keywords:** cellulose-based absorbents, hydrophobization, silanization, oil-water separation, biodegradable materials, environmental sustainability

## Abstract

The need for sustainable, biodegradable materials to address environmental challenges, such as oil-water separation, is growing. Cellulose-based absorbents offer an eco-friendly alternative to synthetic materials. However, their hydrophobicity must be enhanced for efficient application. In this study, cellulose-based sorbents derived from Kraft and half-bleached chemo-thermomechanical pulp (BCTMP) were hydrophobized using silanization and alkyl ketene dimer (AKD) techniques. Hydrophobic properties were successfully imparted using methyltrimethoxysilane (MTMOS), n-octyltriethoxysilane (NTES), and N-(2-Aminoethyl)-3-aminopropyltrimethoxysilane (AATMS), with water contact angles ranging from 120° to 140°. The water sorption capacity was significantly reduced to below 1 g/g, whereas the oil sorption capacity remained high (19–28 g/g). The most substantial reduction in water vapor absorption (3–6%) was observed for the MTMOS- and AATMS-silanized samples. These results demonstrate the potential of hydrophobized cellulose-based sorbents as sustainable alternatives for oil-water separation, contributing to environmentally friendly water treatment solutions.

## 1. Introduction

Water pollution, particularly from oil spills, poses a significant threat to ecosystems, human health, and water resources worldwide [1]. Oil spills, whether from industrial accidents or transportation failures, can cause long-lasting environmental damage, affecting marine and freshwater systems [2]. Although efforts to mitigate oil spills have improved over recent decades, the need for effective, scalable, and environmentally friendly oil removal methods remains critical [3,4]. Traditional methods, such as filtration, sedimentation, and chemical dispersants, are expensive, inefficient, or contribute to secondary pollution [5,6]. Adsorbent materials, which can selectively remove oil from water, offer a promising alternative due to their efficiency, low cost, and potential for recycling the collected oil [7].

Currently, the most used adsorbents are synthetic, often derived from non-renewable sources such as polypropylene. While these materials effectively absorb oil, their non-biodegradable nature creates long-term environmental problems. The accumulation of synthetic sorbents after use leads to additional pollution, counteracting their intended benefits. Consequently, there is a growing interest in developing biodegradable, renewable alternatives that can address both the immediate need for oil removal and the long-term sustainability goals of environmental protection [8,9].

Cellulose, a biopolymer abundantly available from plant sources, is a promising candidate for use as a sustainable sorbent. With annual global production exceeding 1.5 billion tons, cellulose is widely recognized for its biodegradability, renewability, and versatility in various applications [10,11]. Cellulose is a chain polymer composed of repeating anhydroglucose units linked by beta-1-4-glycosidic bonds [11,12,13,14,15]. It is composed of glucose molecules with three reactive -OH hydroxyl groups, a primary one at C6 and two secondary ones at C2 and C3, as well as carboxyl groups -COOH [16,17,18]. The hydroxyl groups play a crucial role in the structure and properties of cellulose. They can be modified by substituting with different functional groups to enhance cellulose's properties and expand its range of applications [19,20]. These modifications can improve the solubility, thermal stability, biodegradability, and mechanical properties of cellulose derivatives, as well as provide unique functionalities, such as antimicrobial properties, water repellency, and flame retardancy, depending on their specific functions. However, natural cellulose has inherent limitations in oil-water separation. Thus, chemical modification of cellulose to impart hydrophobic properties is necessary to enhance its performance in oil-water separation applications.

Hydrophobization of cellulose can be achieved through various chemical methods, including silanization and alkyl ketene dimer (AKD) treatment. These methods modify the surface properties of cellulose, increasing its water repellency while maintaining its ability to absorb non-polar substances such as oils [21,22,23,24]. Previous studies have shown that hydrophobic cellulose-based materials can achieve high efficiency in separating oil from water; however, there is still a need to optimize these methods for different types of cellulose and hydrophobizing agents to improve selectivity, stability, and environmental compatibility.

In this study, we aim to explore the hydrophobization of cellulose-based sorbents derived from Kraft and half-bleached chemo-thermomechanical pulp (BCTMP) using silanization and AKD techniques. By employing different types of silanes—methyltrimethoxysilane, n-octyltriethoxysilane, and N-(2-Aminoethyl)-3-aminopropyltrimethoxysilane—we investigate their effectiveness in imparting hydrophobic properties to cellulose. The hydrophobized cellulose-based sorbent was thoroughly tested to evaluate the effectiveness of the selected hydrophobization methods. The water contact angle and sorption capacity measurements were conducted to assess the hydrophobicity of the samples. A moisture content test was conducted to measure the extent of moisture absorption from the air. Furthermore, the density and porosity of the samples were analyzed to understand the structural characteristics that influence their sorption capacities.

This research contributes to the growing body of knowledge on sustainable materials for environmental remediation, focusing on developing biodegradable and efficient alternatives to synthetic oil sorbents. By enhancing the hydrophobicity of cellulose, we aim to provide a renewable solution to the global challenges of oil spills and water pollution.

## 2. Results and Discussion

### 2.1. Density and Porosity

The averaged results of density and porosity analysis are presented in Table 1. The density of cellulose-based sorbent produced from 2 wt% cellulose pulp ranged from 28 to 44 kg/m^3^, while the average porosity was 97 ± 0.2%. These differences may have originated during the manufacturing stage of the materials. The pulps were composed of deionized water and fibers, intentionally excluding gelling and stabilizing agents, to ensure that no additional substances influenced the sorption capacity of the samples. Consequently, despite using the same pulp volume per sample, slight variations in the water-fiber ratio may have occurred. Although the morphology of the samples is not the primary focus of this research and will be addressed in future studies, the authors assert that understanding the porosity is crucial for assessing the sorption capacity. Porosity, which encompasses the volume and number of pores, positively influences the sorption capacity of a material. This correlation is well-supported by numerous reports [7,9,25,26,27,28].

### 2.2. Water Contact Angle

WCA measurements were conducted on both the hydrophobized and control samples. Measurements were taken at 8 random locations on the samples, including surface and cross sections. Subsequently, the results were averaged and the standard deviation was calculated. For the control samples, the droplets were immediately absorbed upon application, resulting in a recorded angle of 0° (Table 2). For the hydrophobized samples, the contact angle ranged from 120° to 140°, indicating that all samples could be considered hydrophobic because the angle exceeded 90°. The table also shows the grouping of results according to the statistically significant differences between them, as determined by the Tukey test (*p* ≤ 0.05). The same letters in the groups between each sample code indicate no significant difference. Significant differences can be found between the codes of samples that do not include the letter ‘a’ in their group, and these samples had noticeably lower WCA than samples that contained this letter in their group.

**Figure 1:**
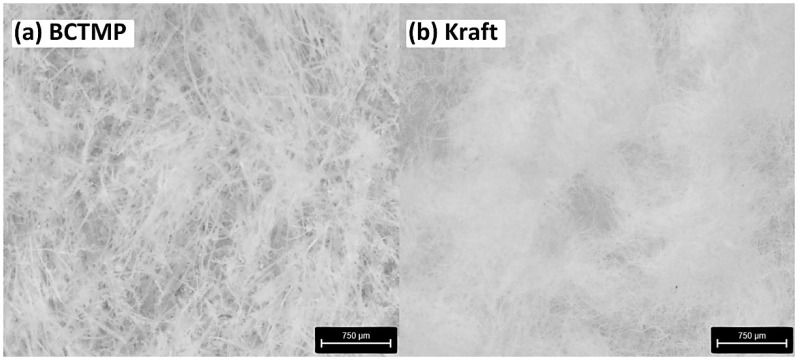
Microscopic images of (**a**) BCTMP and (**b**) Kraft control samples cross-section.

The relationship between the cellulose type and contact angle reduction over time can be observed in a small manner (Table 2). According to the presented results, samples containing BCTMP cellulose showed a more significant reduction in the contact angle than the Kraft samples. This observation could be attributed to the closed pores of Kraft cellulose samples. In contrast, the larger spaces between the fibers in the BCTMP cellulose samples allowed the water droplets to collapse between the fibers, leading to a reduction in the angle. Figure 1 displays microscopic images of BCTMP and Kraft cellulose control sample cross-sections, highlighting structural disparities and fiber gaps. The nature of the sorption property can be influenced by various factors, including the type of pulp utilized and the extent of chemical or mechanical pulping of the fibers [29]. As illustrated, the Kraft cellulose samples exhibit a more compact pore structure than BCTMP samples. This structure results from the Kraft pulp-obtaining process, which involves the chemical dissolution of the pulp to remove lignin, followed by pressing and drying at elevated temperatures. This results in the closure of most pores, with some remaining closed even when re-saturated [30,31,32]. This issue was more pronounced in BCTMP pulp samples but was also observed in all variants.

One of the objectives of this study was to produce samples with high porosity, averaging 97%. The fluctuating standard deviation values indicate that the high porosity may have caused droplets to fall into gaps between fibers, resulting in false lowering of the WCA values. The contact angle values reported in this study were consistent with previous research findings. The hydrophobization of cellulosic materials with MTMOS vapors results in contact angles ranging between 100° and 120° [25,33], while studies employing AKD have achieved values greater than 140° [34,35]. Notably, the higher contact angles obtained by Li et al. (2021) may be attributed to using higher crosslinking temperatures of AKD in their study, leading to more efficient hydrophobization [35]. The selection of silanes was motivated by Nowak et al. (2022), who used silanes as mentioned above for hydrophobized paper [36]. In these studies, the contact angles were approximately 110° for MTMOS, 105° for NTES, and 60° for AATMS. These values are lower than those obtained in this study, indicating that vapor silanization is more effective, despite the much larger specific surface area of the samples being hydrophobized.

### 2.3. Sorption Capacity

**Figure 2:**
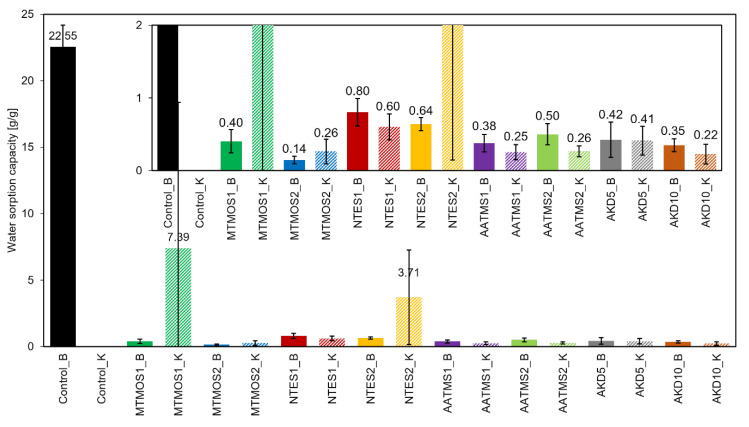
Results of sorption capacity of cellulose-based sorbents to water.

The hydrophobization process was largely successful for most modification variants, significantly reducing the water absorption from 22.55 g/g of material to less than 1 g/g in the case of BCTMP cellulose, such as MTMOS2_B (Figure 2). In contrast, for the Kraft control samples (Control_K), it was not feasible to measure the sorption capacity because the samples lost their structural integrity upon immersion in water, resulting in fiber dispersion within the beaker. Hydrophobization enabled these samples to retain their structure in water with a sorption capacity of less than 1 g/g. MTMOS1_K and NTES2_K showed only partial hydrophobization, leading to higher absorbency than other hydrophobized variants but lower than the control samples, which disintegrated in water. This may be due to an insufficient amount of hydrophobizing agents or uneven silane distribution in the vapor phase. The detailed water sorption capacity results are summarized in Figure 2.

The results of the WCA demonstrated the hydrophobic nature of all the samples, with angles exceeding 90°. However, WCA was measured only for the surface properties of the sample. In contrast, the sorption capacity measurements encompassed the entire sample, rendering the latter a more comprehensive and accurate assessment for this research. These observations suggest the presence of non-hydrophobic regions within the samples, which may have contributed to water absorption in the two partially hydrophobized variants. The probable reason for the observed differences in the sorption test was the different porosity of treated and untreated cellulose. Similar observations and conclusions were reported by Perdoch et al. (2024) and Chang et al. (2018), in which the authors described how water transport through the paper is related to the characteristics of the internal pores of the cellulose fibers [19,37]. Moreover, silicone–cellulose interactions can play a critical role by influencing the fiber saturation point and moisture content of the material. The swelling process in the wet stage uncovered new active sorption sites (OH groups), which were not available for modification in the dry stage owing to the steric effect. Consequently, during a swelling process, new hygroscopic areas are activated [29]. The lowest sorption capacity, indicating the most effective hydrophobization, was achieved with variants hydrophobized with higher amounts of MTMOS and AKD, specifically MTMOS2_B/K and AKD10_B/K. The WSC values for these variants were below 0.3 g/g of material.

Regarding the relationship between the hydrophobizing agents employed, significant differences were observed between the variants hydrophobized with different amounts of MTMOS silane. The amount of silanes (0.8 g/g of sample) used for MTMOS1_B/K samples appeared insufficient to achieve complete hydrophobicity. For the AKD variants, a concentration of 10% yielded superior results compared with 5%, although the differences were not as marked as those observed with MTMOS. No significant differences among the other variants were noted for the different amounts of hydrophobizing agents used. Excluding the two variants in which hydrophobization was unsuccessful, the NTES variants exhibited the highest WSC. Consequently, it can be inferred that NTES was the least effective silane, although the material still demonstrated hydrophobic properties.

**Figure 3:**
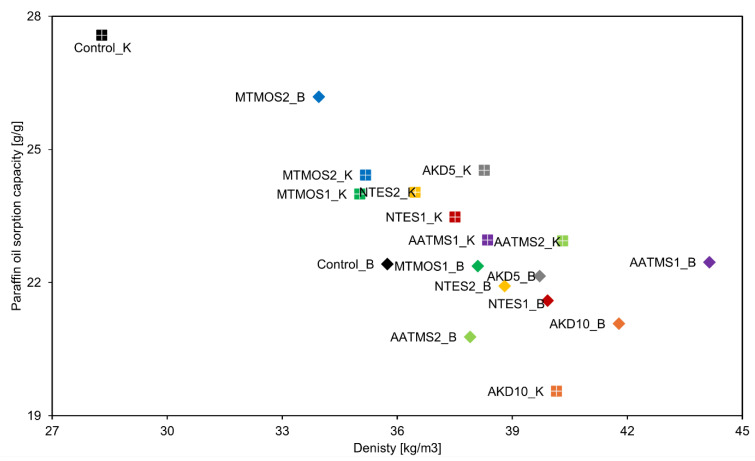
Results of sorption capacity of cellulose-based sorbents to paraffin oil.

Figure 3 presents the results of the sorption capacity tests for paraffin oil, illustrating the correlations between the type of cellulose, hydrophobizing agent, and amounts used. Upon analyzing the results depicted in the figure, a weak correlation was noted between OSC and the type or amounts of the hydrophobizing agent used. As previously discussed, density significantly affects the sorption properties of a given material. The more porous (lower density) the material, the higher its sorption capacity [7,9,25,26,27,28]. Therefore, the observed differences in the OSC can be primarily attributed to variations in the density of the individual samples. In most variants, the OSC oscillated between 19 and 25 g/g of the material. The exception was the Kraft cellulose control samples (Control_K), which had an OSC of more than 27 g/g of material as they had a lower density than the samples of the other variants. Other researchers obtained similar results, e.g., Calcagnile et al. (2017), studying the sorption of, among other things, paraffin oil by cellulosic material, obtained an OSC of about 22 g/g of material [38]. On the other hand, Korhonen et al. (2011) obtained a cellulosic material with a lower density (20–30 kg/m^3^) and a higher OSC, about 30 g/g of material [39].

### 2.4. Moisture Content after Moist Air Exposure

**Figure 4:**
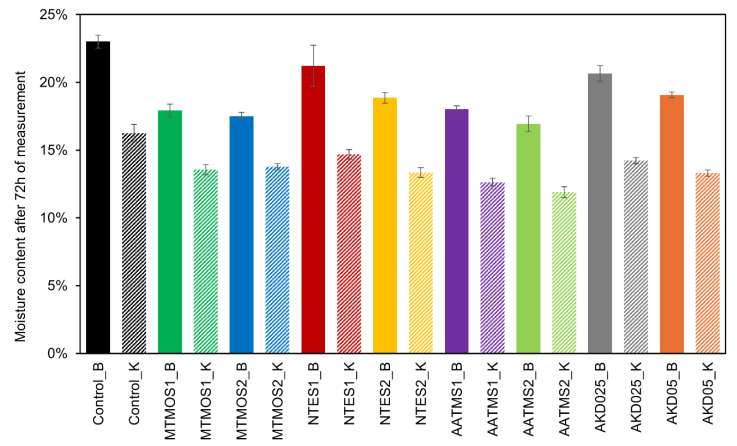
Results of the moisture content of cellulose-based sorbents after 72 h of measurement.

BCTMP control samples (Control_B) reached the highest moisture content, approximately 23%. The MTMOS-hydrophobized samples (MTMOS1_B and MTMOS2_B) exhibited relatively low moisture contents of approximately 17–18%, with no substantial difference between the different amounts of silane used for hydrophobization. NTES1_B and NTES2_B samples demonstrated a noticeable variation in moisture content depending on the amount of NTES used: NTES1_B achieved a moisture content of approximately 21%, whereas NTES2_B showed a moisture content of about 18%. Samples hydrophobized with AATMS silane also exhibited low moisture content after 72 h, with values varying according to the amounts of silane used for hydrophobization. Specifically, AATMS1_B (0.8 g/g) reached a final moisture content of approximately 18%, while AATMS2_B (1.6 g/g) was only 16%. Samples treated with AKD showed a similar trend, with final moisture contents of 20% (AKD5_B) and 19% (AKD10_B), respectively. Detailed moisture content results are presented in Figure 4.

Kraft cellulose control samples (Control_K), similar to the BCTMP control samples (Control_B), had the highest moisture content at approximately 16%. The MTMOS silane samples (MTMOS1/2_K) displayed lower values of approximately 3% and 13%. As observed for the BCTMP cellulose samples, no significant impact of the amount of silanes used for hydrophobization on the moisture content was noted for the MTMOS samples. The NTES1_K and NTES2_K samples showed slight differences in moisture content: NTES1_K had a moisture content of approximately 14%, whereas NTES2_K had a moisture content of approximately 13%. AATMS silane-treated samples had the lowest moisture content after 72 h, with values varying according to the amounts of silanes used for hydrophobization. For AATMS1_K, the moisture content was approximately 12%, while for AATMS2_K it was around 11%. AKD-hydrophobized samples had moisture contents of 14% (AKD5_K) and 13% (AKD10_K).

In summary, both control samples (Control_B and Control_K) exhibited the highest moisture content, indicating that hydrophobization effectively reduces the moisture content of the cellulose samples. The lowest moisture content was observed in the samples hydrophobized with AATMS silane and MTMOS1/2_B, indicating that these were the most effective silanes in this study. Generally, the amounts of agents used for hydrophobization influenced the moisture content, except for samples modified with MTMOS silane. Throughout the study, Kraft cellulose samples absorbed less water vapor, likely because of the closed pore structure of cellulose [29], which the authors believe is advantageous for the potential use of this material compared to BCTMP cellulose samples. In oil sorption capacity tests, Kraft cellulose samples demonstrated a higher sorption capacity than BCTMP cellulose, while in moisture content tests, they absorbed less water vapor than BCTMP. This characteristic is potentially beneficial for storing materials without compromising their sorption potential.

Previous studies have also explored reducing moisture content in various cellulosic materials by applying hydrophobic substances. For instance, Mazela et al. (2022) and Majka et al. (2023) employed MTMOS silane as a starch coating with a silane additive [40,41]. However, these studies did not achieve a reduction in the equilibrium moisture content, which exceeded that of the control samples. In subsequent experiments, Perdoch et al. (2024) successfully incorporated NTES silane into pulp during the paper sheet manufacturing process, significantly reducing the equilibrium moisture content [19].

## 3. Materials and Methods

### 3.1. Materials

Two types of cellulose were used to develop the cellulose-based absorbents: Kraft and BCTMP. The Kraft pulp fibers were made from 100% eucalyptus using the elemental chlorine-free (ECF) bleaching process and came in the commercial form of Guaíba Pulp, Rio Grande do Sul, Brazil. The brightness was 89 + % (ISO 2470 [42]), dirt count ≤ 2.5 mm^2^/kg a.d. (ISO 5350-2 [43]), viscosity ≥ 700 mL/g (ISO 5351 [44]), and pH ≥ 5.0 (ISO 6588-1 [45]). The pure spruce BCTMP used was purchased from Waggeryd Cell AB, Vaggeryd, Sweden. The brightness was 65 + %, drainage resistance was 22° SR (ISO 5267-1 [46]), and fiber length was 1.7 mm. Additionally, methyltrimethoxysilane [OSF Silane 6070, Biesterfeld, Lügde, Germany], n-octyltriethoxysilane [BSC Silane MX041, Biesterfeld, Lügde, Germany], N-(2-Aminoethyl)-3-aminopropyltrimethoxysilane [BSC Silane A020, Biesterfeld, Lügde, Germany], and alkyl ketene dimer [BIM IS 9335, BIM Kemi AB, Stenkullen, Sweden] were utilized for the hydrophobization of the manufactured materials. Deionized water and paraffin oil (CAS 8012-95-1, AKTYN, Suchy Las, Poland) were used for water/oil sorption capacity tests.

### 3.2. Material Preparation

#### 3.2.1. Manufacturing of Cellulose-Based Absorbents

**Figure 5:**
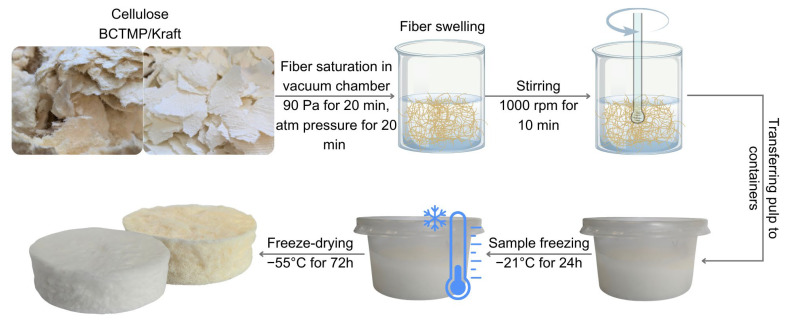
Schematic representation of the cellulose-based absorbent manufacturing process.

Kraft or BCTMP cellulose was placed in a beaker containing deionized water, with the fiber concentration set at 2% (*w*/*v*), then the fibers were saturated to the maximum with water in a vacuum chamber (Goldbrunn 450, Goldbrunn, Oberstdorf, Germany). The vacuum (90 Pa) was maintained for 20 min, and then the fibers were left at atmospheric pressure for 20 min to swell. The fiber suspension was then stirred with a mechanical stirrer (CAT R18, CAT, Mannheim, Germany) at 1000 rpm for 10 min to break up the fiber clusters and obtain a more homogenous pulp. The pulp was distributed into 80 mL polypropylene cylindrical containers, frozen (−21 °C) for 24 h, and lyophilized in a freeze-dryer (LabTech HyperCOOL HC3055, LabTech, Sorisole, Italy) at −55 °C (1.3 × 10^−1^ Pa) for 72 h. The manufacturing process of cellulose-based absorbent samples is shown in Figure 5.

#### 3.2.2. Silanization of Cellulose-Based Absorbent

Silanization was carried out using the method often described in the literature [9,25,33] of infusing material samples with a concentrated silane solution. For this purpose, the solution was placed at the bottom of a large beaker, and cellulose-based absorbents were placed above it on a separating mesh. For each type of cellulose-based absorbent, two amounts of silane were used (0.8 and 1.6 g/g of sample) to test the correlation between the amount of silane infused and silane hydrophobization efficiency. The beaker was tightly sealed with foil and placed in a drying oven at 70 °C for 48 h. After hydrophobization was completed, the samples were placed for 24 h in a double-bottomed container with distilled water at the bottom to hydrolyze the alkoxy group, as even small amounts of water can catalyze this reaction. According to literature [47,48], a hydrolysis reaction is necessary for a permanent and stable modification effect. For convenience, samples infused with less silane (0.8 g/g of sample) are labeled as 1 in the sample code, and samples infused with more silane (1.6 g/g of sample) are labeled as 2, for example, MTMOS1_B, MTMOS2_B (Table 1).

#### 3.2.3. Alkyl Ketene Dimer Hydrophobization of Cellulose-Based Absorbent

**Figure 6:**
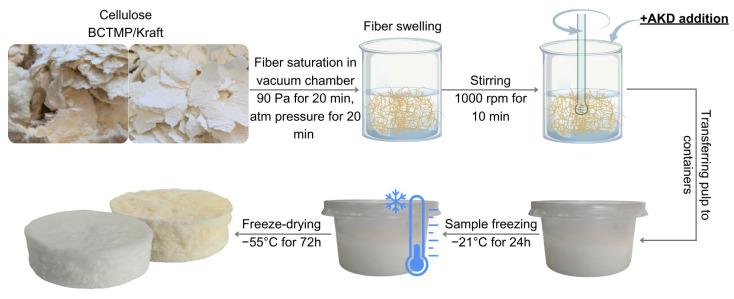
Schematic representation of cellulose-based absorbent manufacturing process with alkyl ketene dimer addition.

AKD hydrophobization took place during the production of the cellulose-based absorbent. During the mixing stage of the pulp with a mechanical mixer, AKD was added to the pulp at two concentrations: 5 or 10 wt% (to the dry weight of fibers). The samples then underwent a thermo-crosslinking process at 105 °C for 24 h. The manufacturing process of cellulose-based absorbent samples hydrophobized with AKD is shown in Figure 6. A detailed explanation of the manufactured cellulose-based sorbents (sample codes) is presented in Table 3.

### 3.3. Density and Porosity

The density (*ρ*) was calculated from the weight (*m*) and volume (*V*) of the samples. The height and diameter of each sample were then measured. The porosity was calculated using the following formula:

$$\rho =\frac{m}{V\;}\;[\frac{kg}{m^{3}}]$$

$$Porosity=(1-\left(\frac{\rho }{\rho_{c}}\right))\times 100\%\;[\%]$$

where *ρ* is the calculated density, and *ρ_c_* is the theoretical density, which, according to the literature, is 1.5 g/cm^3^ [49].

### 3.4. Microscopic Imaging of Cross-Sections of Protein Materials

The cross-sectional areas of the produced cellulose samples were examined using a digital microscope (Leica Microsystems Emspira 3, Wetzlar, Germany) (Figure 1). Images were processed using the LAS X program (Leica Application Suite X; Leica Microsystems, Wetzlar, Germany).

### 3.5. Water Contact Angle (WCA)

The hydrophobicity of the cellulose samples was assessed using WCA measurements. Employing a DSA 25 KRÜSS goniometer from Kruss Scientific GmbH in Hamburg, Germany, the sessile droplet contact angle was determined at a temperature of 20 °C and air relative humidity of 50%. A 0.07 μL water droplet was applied to the sample surface, and an image of the droplet was captured after 1 min. Subsequent measurements were taken at 60 s intervals for 5 min. This measurement protocol was repeated eight times for each variant.

### 3.6. Sorption Capacity

For the sorption capacity (SC) test, the cellulose-based sorbents were cut into 4 samples using a bandsaw. The sorption capacities of deionized water and paraffin oil were determined. The values were calculated as the ratio of the absorbed water/paraffin oil to the initial mass of cellulose material. Subsequently, the weighed samples were immersed in a beaker containing 50 mL of substance for 15 min, removed, and left on a mesh to drain the excess substance for 1 min. The specimens were weighed, and the water (WSC) and oil sorption capacity (OSC) were computed using the prescribed formula, where *m*_1_ is the mass of the sample after the test and *m*_0_ is the mass before the test:

$$SC=\frac{m_{1}-m_{0}}{m_{0}}\;[\frac{g}{g}of\;sample]$$

### 3.7. Moisture Content after Moist Air Exposure

The moisture content of the samples was measured to assess the extent of moisture absorption from the moist air. The cellulose-based sorbents were sectioned into four equal parts using a bandsaw. Eight samples were prepared for each variant. The samples were then dried to a constant weight at 105 °C for 24 h. After cooling to room temperature in a desiccator, they were placed in tightly sealed containers over a supersaturated ammonium monophosphate solution to ensure a relative humidity of 90–95%. Moisture absorption by the samples was monitored by measuring weight gain after 72 h.

### 3.8. Statistical Analysis

The analysis of the oil sorption capacity results included one-way ANOVAs. Significant parameters identified in the ANOVAs were subjected to further analysis using post-hoc Tukey’s test. Results were deemed statistically significant at a level of *p* < 0.05. R (Version 4.3.2) and RStudio (Version 2023.09.1+494) were used for all the statistical analyses.

## 4. Conclusions

This study successfully demonstrated the hydrophobization of cellulose-based materials derived from Kraft and BCTMP cellulose using silanization and alkyl ketene dimer (AKD) techniques. Hydrophobic properties were confirmed by water contact angles ranging from 120° to 140°, and the water sorption capacity was significantly reduced while maintaining a high oil sorption capacity. The most effective results were obtained with higher concentrations of methyltrimethoxysilane (MTMOS) and AKD, achieving minimal water absorption (below 0.3 g/g) and a significant reduction in moisture content (3–6%). The density of the materials played a key role in determining their sorption capacity, underlining the importance of optimizing both chemical modification and physical properties for real-world applications. In conclusion, MTMOS emerged as the most effective hydrophobizing agent, followed by AATMS and AKD.

The hydrophobized cellulose-based sorbents developed in this research offer a promising, biodegradable alternative to synthetic sorbents for oil-water separation. Their potential applications extend beyond oil spill remediation to broader environmental sustainability efforts, where efficient and eco-friendly materials are needed to address pollution. These materials contribute to the growing field of sustainable solutions for environmental challenges, providing a renewable, decomposable option for water treatment. Future research should focus on scaling these findings for industrial use and further explore the mechanical and structural properties of the hydrophobized cellulose-based sorbents.

## Figures and Tables

**Table 1:** Summary of the density and calculated porosity results of cellulose-based absorbents.

Sample Code	Density [kg/m^3^]		Porosity [%]	
	Average	±SD *	Average	±SD
Control_B	35.74	3.57	97.62	0.24
Control_K	28.29	2.92	98.11	0.19
MTMOS1_B	38.10	4.83	97.46	0.32
MTMOS1_K	35.02	3.16	97.67	0.21
MTMOS2_B	33.95	2.92	97.74	0.19
MTMOS2_K	35.17	3.11	97.66	0.21
NTES1_B	39.92	2.82	97.34	0.19
NTES1_K	37.51	2.44	97.50	0.16
NTES2_B	38.80	1.97	97.41	0.13
NTES2_K	36.46	2.21	97.57	0.15
AATMS1_B	44.14	1.78	97.06	0.12
AATMS1_K	38.35	2.18	97.44	0.15
AATMS2_B	37.90	1.34	97.47	0.09
AATMS2_K	40.31	2.05	97.31	0.14
AKD5_B	39.71	2.60	97.35	0.17
AKD5_K	38.26	4.67	97.45	0.31
AKD10_B	41.78	3.04	97.21	0.20
AKD10_K	40.15	3.30	97.32	0.22

* SD—Standard deviation

**Table 2:** Compilation of the results of the contact angle of cellulose-based sorbents at the 1st and 5th minute of measurement, their standard deviation, and the reduction of WCA through time.

Sample Code	Water Contact Angle [°]				Reduction of WCA through Time [°]
	Elapsed Time from Drop Application [min]				
	1	±SD	5	±SD	
Control_B	0 ^e^ *	0	0	0	0.00
Control_K	0 ^e^	0	0	0	0.00
MTMOS1_B	122.01 ^c^	7.33	116.22	6.59	−5.78
MTMOS1_K	131.86 ^abcd^	8.89	129.53	9.17	−2.32
MTMOS2_B	127.08 ^bcd^	6.22	123.88	7.06	−3.20
MTMOS2_K	132.48 ^abcd^	7.45	130.39	7.37	−2.09
NTES1_B	131.10 ^abcd^	6.23	124.45	10.68	−6.65
NTES1_K	131.73 ^abcd^	2.91	129.73	2.71	−2.00
NTES2_B	134.64 ^abd^	6.71	130.50	6.92	−4.15
NTES2_K	130.13 ^abcd^	7.43	127.86	6.88	−2.27
AATMS1_B	128.01 ^abcd^	13.06	123.29	12.18	−4.72
AATMS1_K	138.83 ^a^	5.01	136.16	5.29	−2.67
AATMS2_B	127.13 ^abcd^	9.47	124.12	9.62	−3.01
AATMS2_K	123.44 ^bc^	7.49	122.19	10.56	−1.25
AKD5_B	133.91 ^abd^	5.71	131.96	6.59	−1.95
AKD5_K	138.56 ^ad^	4.79	137.03	5.28	−1.53
AKD10_B	134.89 ^abd^	3.11	133.17	3.92	−1.72
AKD10_K	135.70 ^ad^	5.07	134.02	5.34	−1.68

* results groups determined by Tukey test (*p* ≤ 0.05).

**Table 3:** Explanation of the sample codes.

Sample Code	Hydrophobizing Agents	Amount of Hydrophobizing Agents Added		Cellulose Type Used (Concentration 2 wt%)
Control_B	(Control sample)	-		BCTMP
Control_K	(Control sample)	-		Kraft
MTMOS1_B	MTMOS	0.8	g/g of sample	BCTMP
MTMOS1_K	MTMOS	0.8		Kraft
MTMOS2_B	MTMOS	1.6		BCTMP
MTMOS2_K	MTMOS	1.6		Kraft
NTES1_B	NTES	0.8		BCTMP
NTES1_K	NTES	0.8		Kraft
NTES2_B	NTES	1.6		BCTMP
NTES2_K	NTES	1.6		Kraft
AATMS1_B	AATMS	0.8		BCTMP
AATMS1_K	AATMS	0.8		Kraft
AATMS2_B	AATMS	1.6		BCTMP
AATMS2_K	AATMS	1.6		Kraft
AKD5_B	AKD	5 wt%	to the dry weight of fibers	BCTMP
AKD5_K	AKD	5 wt%		Kraft
AKD10_B	AKD	10 wt%		BCTMP
AKD10_K	AKD	10 wt%		Kraft

## Data Availability

The data used to support the findings of this study can be made available by the corresponding authors upon request.
